# MODERATING THE EFFECTS OF COGNITIVE DECLINE ON DAILY FUNCTIONING IN OLDER ADULTS: THE ROLE OF SOCIAL SUPPORT

**DOI:** 10.1093/geroni/igae098.3214

**Published:** 2024-12-31

**Authors:** Youngmin Cho, Bei Wu, Baiming Zou, Lixin Song, Ruth Anderson, Anna Beeber

**Affiliations:** Johns Hopkins University, Baltimore, Maryland, United States; New York University, New York, New York, United States; University of North Carolina, Chapel Hill, North Carolina, United States; University of Texas Health Science Center at San Antonio, San Antonio, Texas, United States; Duke University, Durham, North Carolina, United States; Johns Hopkins University, Baltimore, Maryland, United States

## Abstract

As cognitive impairments increasingly affect community-dwelling older adults (CDOA), it becomes significant to understand the longitudinal impact of social support factors—such as living arrangements, social network size, and participation in social activities—on the relationship between cognitive status and daily functioning. Daily functioning includes activities of daily living (ADL), instrumental activities of daily living (IADL), physical capacity, and mobility. This study aims to determine whether social support factors moderate the relationship between cognitive status and daily functioning. We analyzed data from the National Health and Aging Trends Study (NHATS), a longitudinal survey of a nationally representative cohort of Medicare beneficiaries aged 65 and over in the US. Our analysis included eight years of data from 6,238 community-dwelling participants. Using linear mixed-effects models, we explored the interaction effect between cognitive status and social support factors on trajectories of daily functioning. Overall, all areas of daily functioning significantly declined over time, with faster decreases observed as cognitive status worsened. Living arrangements moderated the relationship between cognitive status and IADL, specifically mitigating the negative impact of cognitive decline on IADL. Increased participation in social activities eased the decline in ADL for individuals with probable dementia and in physical capacity for those with mild cognitive impairment (MCI); however, the size of the social network had no significant impact. Our findings highlight the importance of incorporating social support factors into tailored interventions to mitigate declines in daily functioning among CDOA, tailored to their specific cognitive status.

